# A new flavor of cellular Atg8-family protein lipidation – alternative conjugation to phosphatidylserine during CASM

**DOI:** 10.1080/15548627.2021.1947730

**Published:** 2021-07-12

**Authors:** Joanne Durgan, Oliver Florey

**Affiliations:** Signalling Programme, Babraham Institute, Cambridge, UK

**Keywords:** ATG8, ATG4, lipidation, LC3-associated phagocytosis, endolysosome, phosphatidylserine, non-canonical autophagy

## Abstract

Atg8-family protein lipidation is the most commonly used marker for monitoring autophagy. During macroautophagy, Atg8-family proteins are specifically conjugated to phosphatidylethanolamine (PE) in forming, double-membrane autophagosomes. A distinct, non-canonical autophagy pathway also operates, characterized by the Conjugation of ATG8s to endolysosomal Single Membranes (CASM). In our new study, we show that CASM is associated with the alternative conjugation of Atg8-family proteins to phosphatidylserine (PS), and PE, in response to various cellular stimuli. We also discover differences in the regulation of conjugation to PE and PS by ATG4s, and altered dynamics between the two species. The identification of alternative Atg8-family protein PS lipidation opens up exciting new questions on the roles, regulation and biology of Atg8-family proteins during non-canonical autophagy.

## Main

The Atg8 (autophagy related 8) family of ubiquitin-like proteins are key to the formation and cargo loading of phagophores, and the maturation of double-membrane autophagosomes. During autophagy, Atg8-family proteins undergo a unique post-translational modification, orchestrated by two ubiquitin-like conjugation systems, through which the C-terminal glycine is covalently linked to a phospholipid head group. Atg8-family protein lipidation is associated with a distinctive protein band shift between lipidated and unlipidated forms (SDS-PAGE and western blotting), and cellular relocalization of Atg8-family proteins from cytosol to membranes (detected by microscopy). These Atg8-family protein characteristics have become widely used for monitoring autophagy.

Ground-breaking work from the Ohsumi lab used mass spectrometry to identify phosphatidylethanolamine (PE) as the sole target for Atg8 lipidation during canonical autophagy; subsequent studies confirmed the formation of Atg8–PE and Atg8-family protein conjugation to PE in both yeast and mammalian cells. Interestingly, a second lipid, phosphatidylserine (PS), also bears the amino group required for Atg8 lipidation, and can be conjugated to Atg8 *in vitro*. However, Atg8–PS has never been identified in cells, and it was speculated that this modification is actively inhibited by cellular pH, phospholipid composition or the presence of a regulatory factor. The cellular exclusivity of Atg8–PE conjugation has become well established in the autophagy field.

A parallel non-canonical autophagy pathway has since been discovered that utilizes some components of the autophagy machinery to conjugate Atg8-family proteins to single-membranes (CASM) of the endolysosomal system. This pathway is independent of nutrient status and upstream initiation factors (ULK-ATG13-RB1CC1), but dependent on the core, ubiquitin-like conjugation machinery. An important example of non-canonical autophagy is LC3-associated phagocytosis (LAP), where LC3 lipidation to phagosomes supports efficient killing and/or clearance of pathogens or apoptotic cells, with important biological consequences. CASM also occurs in response to TRPML1 and STING1 agonists, pharmacological disruption of ion balance, and various engulfment events, including endocytosis, macropinocytosis and entosis. In all literature to date, it has been assumed that PE is the only lipid conjugated to Atg8-family proteins during these non-canonical autophagy processes.

**Figure 1.**
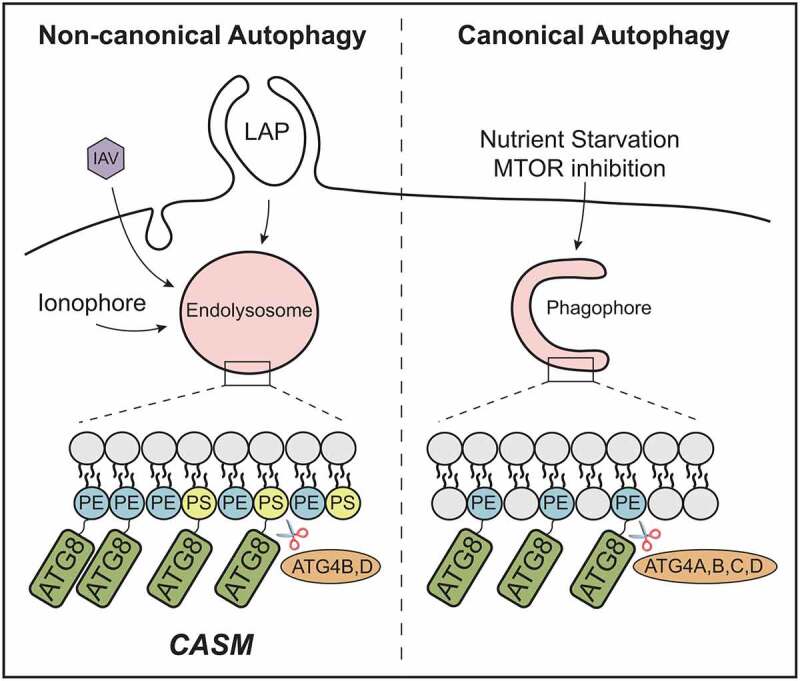
CASM promotes Atg8-family protein (ATG8) conjugation to PE and PS. Activation of non-canonical autophagy through ionophores, influenza A virus (IAV) and LC3-associated phagocytosis (LAP), promotes the conjugation of Atg8-family protein isoforms to both PE and PS in single-membrane endolysosomes. In contrast, only LC3/GABARAP–PE is found in double-membrane autophagosomes. ATG4 isoforms differentially delipidate LC3/GABARAP–PE and LC3/GABARAP–PS species

In our recent study [1], we considered the fact that distinct autophagy-related pathways target different membranes, and therefore revisited the analysis of Atg8-family protein lipidation. By combining CRISPR and pharmacological approaches, robust and specific activation of either canonical or non-canonical autophagy was induced. To stimulate the canonical pathway, wild-type cells were treated with an MTOR inhibitor; to activate non-canonical autophagy, autophagosome-deficient *ATG13*^−/-^ cells were treated with monensin. We then enriched for GFP-tagged Atg8-family proteins and used mass spectrometry to identify the conjugated lipid. In agreement with previous findings, we confirmed the sole presence of LC3/GABARAP–PE during canonical autophagy. Importantly though, during monensin-induced CASM, we detected LC3/GABARAP–PS (and LC3/GABARAP–PE), providing the first evidence for alternative Atg8-family protein lipidation in cells. This held true for all isoforms of Atg8-family proteins. We also demonstrated induction of LC3/GABARAP–PS during more physiologically relevant non-canonical autophagy processes, including LAP and influenza infection, suggesting this represents a common feature of CASM (Figure 1).

The alternative conjugation of Atg8-family proteins during CASM is consistent with the fact that phosphatidylserine is enriched at endolysosomal membranes and plasma membrane (inner leaflet). Indeed, using a PS sensor (RFP-LactC2), we found that membranes targeted for CASM were clearly PS positive, while the sensor was not detectable at forming autophagosomes. The exact lipid composition of autophagosomes is still not known. Recent data suggest they do contain some PS, though perhaps at insufficient levels for detection by our sensor. The differential formation of LC3/GABARAP–PS may thus reflect simple differences in PS abundance at target membranes, though additional regulatory mechanisms may exist. For instance, subcellular conditions may influence the efficiency of LC3/GABARAP PE/PS conjugation at autophagosomes versus endolysosomes (e.g., local pH, phospholipid composition or degree of membrane curvature). Alternatively, differences in ATG4-mediated delipidation may contribute. Further work will be needed to address this important question.

ATG4s are dual-specificity proteases, which prime Atg8-family proteins for lipidation by exposing the C-terminal glycine, then drive subsequent delipidation and recycling off membranes. In mammals, the ATG4 family comprises four isoforms (ATG4A to ATG4D). Using the co-crystal structure of LC3B-ATG4B, we explored the possible consequences of PE versus PS conjugation; these lipids differ by just a single carboxyl group. Molecular modeling of each lipid suggested the additional bulk of the PS carboxyl group may sterically hinder catalytically important residues within ATG4B. Consistent with this, using *in vitro* de-lipidation assays, ATG4B (and ATG4A/C) deconjugated LC3–PS less efficiently than LC3–PE. In contrast, ATG4D showed more optimal activity against LC3/GABARAP–PS, pointing to important differences between ATG4 isoforms. Using CRISPR knockout, we confirmed that *ATG4D* loss increases levels of both LC3–PS and LC3–PE, consistent with a new function for this little-studied family member. Interestingly though, in a cellular system, ATG4B could also facilitate LC3/GABARAP–PS deconjugation, suggesting additional complexity *in vivo* that will be interesting to investigate further.

Finally, to establish the overall outcome of these mechanistic differences, the dynamics of LC3–PS versus LC3–PE were assessed. During LAP, the ratio between LC3–PS: LC3–PE clearly increases over time, indicating that LC3–PS is a longer-lived species. These data establish a significant molecular distinction between alternatively lipidated Atg8-family proteins. A key question for the future will be to determine the exact physiological function of LC3/GABARAP–PS. To address this convincingly, more refined tools will be required to manipulate the two lipidated species specifically. In addition to its functional impact, LC3/GABARAP–PS also represents an important new tool, providing a “molecular signature” for the CASM pathway that can enable distinction between canonical and non-canonical autophagy signaling.
